# Impact of Systematic Use of Intracardiac Ultrasound during Transseptal Catheterization in the Electrophysiology Laboratory

**DOI:** 10.3390/jcdd10020062

**Published:** 2023-02-02

**Authors:** Nicola Bottoni, Paolo Donateo, Luca Rossi, Michele Malagù, Luca Tomasi, Fabio Quartieri, Andrea Biagi, Matteo Iori, Giacomo Mugnai, Antonella Battista, Stefano Cló, Michele Brignole, Matteo Bertini

**Affiliations:** 1 Cardiology Unit, S. Maria Nuova Hospital, 42100 Reggio Emilia, Italy; 2 Arrhythmologic Center, Lavagna Hospital, 16033 Lavagn, Italy; 3 Guglielmo da Saliceto Hospital, 29121 Piacenza, Italy; 4 Cardiogy Unit, University Hospital of Ferrara, 8-44124 Cona, Italy; 5 Electrophysiology and Cardiac Pacing, Division of Cardiology, Cardio-Thoracic Department, University Hospital of Verona, 1-37126 Verona, Italy

**Keywords:** ablation, TSC, ICE, complications

## Abstract

Aims: To explore the impact of the use of intracardiac echocardiography (ICE) in the ablation of supraventricular arrhythmias requiring transseptal catheterization (TSC), whilst analyzing the reduction in periprocedural complications and complications specifically related to TSC. Methods: A retrospective multicenter study collecting data from consecutive atrial fibrillation (AF) and supraventricular ablation procedures that required TSC was performed in five Italian centers. Based on physician discretion, TSC was performed with or without ICE. Periprocedural complications, separating all complications from complications directly related to TSC, were collected. Independent predictors of periprocedural complications and TSC-related complications were investigated. Results: A total of 2181 TSCs were performed on 1862 patients at five Italian centers from 2006 to 2021, in 76% of cases by AF ablation and in 24% by ablation of other arrhythmias with a circuit in the left atrium. Overall, 1134 (52%) procedures were performed with ICE support and 1047 (48%) without ICE. A total of 67 (3.1%) complications were detected, 19 (1.7%) in the ICE group and 48 (4.6%) in the no ICE group, *p* < 0.001. A total of 42 (1.5%) complications directly related to TSC: 0.9% in the ICE group and 3.1% in the no ICE group (*p* < 0.001). The independent predictors of all complications were age (OR 1,02 95% C.I 1.00–1.05; *p* = 0.036), TSC with the use of ICE (OR 0.27 95% C.I 0.15–0.46; *p* < 0.001) and AF ablation (OR 2,25 95%C.I 1.05–4.83; *p* = 0.037). The independent predictors for TSC complications were age (OR 1.03 95% C.I 1.01–1.06; *p* = 0.013) and TSC with the use of ICE (OR 0.24 95% C.I 0.11–0.49; *p* < 0.001). Conclusions: ICE reduced periprocedural and TSC-related complications during electrophysiological procedures for ablation of left atrial arrhythmias.

## 1. Introduction

During the last ten years, the use of transseptal catheterization (TSC) in the electrophysiology laboratory has increased, especially in its use in the ablation of the substrate of atrial fibrillation (AF). This technique, although highly effective, is still burdened by complications of around 1–6% [1,2,3,4,5]. To increase the safety and efficacy of TSC, tools such as transesophageal or intracardiac (ICE) echocardiography can be used [6]. Transesophageal echocardiography is generally poorly tolerated, and requires high levels of sedation and a dedicated cardiologist specializing in echo. ICE causes an increase in procedural costs and requires additional echocardiographic skills but is well tolerated by patients without the need for specialist echo cardiologist support. From the data in the literature, ICE during TSC is more used in the US than in other countries [4]; percentages of use do not exceed 15% of cases in the largest register published to date [7]. Previous studies reported that the use of ICE during AF ablation was associated with a lower risk of complication [5]. The aim of the present study was to explore the impact of the use of ICE in the ablation of supraventricular arrhythmias in the left atrium requiring TSC, whilst analyzing the reduction in periprocedural complications and complications specifically related to TSC.

## 2. Methods

In this retrospective multicenter study, we collected data from consecutive AF and supraventricular ablation procedures that required TSC, performed at the various participating centers (full list in Appendix A) for the purpose of ablation in the left atrium. All procedures were performed between 1 January 2006 and 31 December 2021. The study protocol was approved by the local ethics committees.

Based on physician discretion, TSC was performed with or without ICE. The patient population was divided into two groups: patients who underwent the TSC without ICE (no ICE group) and patients who underwent the TSC with ICE (ICE group).

Baseline characteristics of patients, such as age, gender, presence of cardiac disease, left ventricular ejection fraction, left atrial diameter and periprocedural anticoagulation regimen, were collected. We considered as “uninterrupted anticoagulant” the patients in whom no dose (in case of direct oral anticoagulant taken once daily) or only morning dose (in case of direct oral anticoagulant taken twice daily) was withdrawn before the procedure. Regarding patients taking vitamin K antagonist, uninterrupted therapy meant patients therapeutically anticoagulated at the time of the procedure without needing heparin bridge the days before. All acute periprocedural complications during in-hospital follow-ups were recorded. All acute complications related to TSC were recorded. Independent predictors of periprocedural complications and TSC-related complications were investigated. 

### 2.1. Transseptal Catheterization with ICE and without ICE

We used the simplified technique described by De Ponti et al. [8]: two catheters are used as reference points respectively in ‘his’ and coronary sinus, without a pigtail catheter in the aorta, atrial pressure curve was used at physician discretion. Transseptal sheath and dilator were placed over the wire in the superior vena cava, 3 to 4 cm above the cavoatrial junction. At this point, the guidewire was removed and the transseptal needle with the stylet was gently inserted, avoiding pushing the needle over the tip of the sheath. At this point, the needle and the sheath are held in the fingers between 4 and 6 o’clock. The needle and the sheath are then pulled back gradually until the tip engages the fossa ovalis. At this point, the tip of the needle is protruded from the distal part of the dilator and puncture of the atrial septum is performed. 

With ICE, the TSC technique is the same as above with the addition of ICE to confirm engagement of tip of the needle and sheath of fossa ovalis.

The 5 participating centers shared a similar experience, up to 2015 ICE (SOUNDSTAR^®^ Catheter, Biosense Webster or ViewFlexTM Catheter, St. Jude Medical-Abbott) was used only in selected difficult cases at physician discretion. Subsequently, from 2016 onwards, systematic use of the ICE was made in most of the TSC procedures. The effectiveness of TSC was also considered as the ability to complete it with adequate positioning of the catheters in the left atrium. All TSCs were performed directly or under the supervision of an experienced operator, defined by the previous performance of >50 procedures. In the case of double TSC during ablation of atrial fibrillation, a single puncture was considered. TSC through patent foramen ovalis was excluded. The majority of procedures were performed under sedation with midazolam plus morphine/fentanyl while general anesthesia was used in a small minority of selected cases. In terms of learning curve, procedures were divided in the whole cohort in chronological order between 1–100, 101–200 and >200.

### 2.2. Study Endpoints

We collected periprocedural complications, separating all complications from complications directly related to TSC. Periprocedural complications included: vascular access complications (groin and retroperitoneal hematoma, arterio-venous fistula), acute aortic root puncture (with or without requiring pericardiocentesis or cardiac surgery), atrial wall injury (contrast in pericardium, perforation requiring pericardiocentesis), pericarditis and/or pericardial effusion, thromboembolism (transient ischemic attack/stroke, pulmonary embolism), acute coronary syndrome, hemidiaphragm paresis and death.

Periprocedural TSC complications included: acute aortic root puncture (with or without requiring pericardiocentesis or cardiac surgery), atrial wall injury (contrast in pericardium, perforation requiring pericardiocentesis), pericardial effusion, thromboembolism (transient ischemic attack/stroke), acute coronary syndrome and death.

The primary endpoint was the occurrence of periprocedural complications. The secondary endpoint was the occurrence of TSC complications.

### 2.3. Statistical Analysis

Statistical analysis was performed per procedure. The data analysis was descriptive. Continuous variables with normal distribution were expressed as mean ± standard deviation. Categorical variables were expressed as an absolute number and percentage (%). The comparison between continuous variables was performed by t-test. Comparison of proportions was performed by Fisher’s test or chi-square as appropriate. In stepwise logistic regression, the dependent variable was represented by the periprocedural complications and complications related to TSC and the independent variables correspond to the significant clinical characteristics (*p* < 0.05). Statistical analyses were performed with SPSS Statistics version 25.0 (IBM Corporation).

## 3. Results

The characteristics of the study population are listed in Table 1. In particular, 2181 procedures were performed on 1862 patients at five Italian centers from 2006 to 2021, in 76% of cases by AF ablation and in 24% by ablation of other arrhythmias with a circuit in the left atrium (accessory pathway, atrial tachycardias, atypical atrial flutter). Overall, 1134 (52%) procedures were performed with ICE support and 1047 (48%) without ICE. From 2006 to 2015, a total of 993 procedures were performed and ICE was used in only 95 (8.3%) procedures, whereas from 2016 to 2021 a total of 1188 procedures were performed, and ICE was used in 1039 (91.6%) procedures.

**Figure 1:**
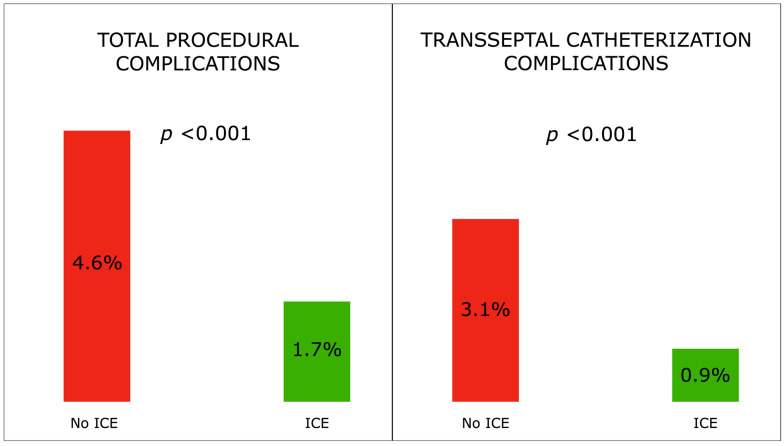
Total and transseptal catheterization-related procedural complications with and without ICE guide.

A total of 67 (3.1%) complications were detected, 19 (1.7%) in the ICE group and 48 (4.6%) in the no ICE group, *p* < 0.001. No procedure-related deaths were observed. A total of 42 (1.5%) complications directly related to TSC: 0.9% in the ICE group and 3.1% in the no ICE group (*p* < 0.001) (Figure 1). In particular, an accidental puncture of the aortic root occurred in a total of 8 patients (0.3%), in 0 (0%) in the ICE group and 8 (0.8%) in the no ICE group, *p* = 0.003; 4 of 8 patients experiencing aortic puncture received emergency cardiac surgery; accidental puncture of the atrial wall was seen in a total of 14 (0.6%) patients, 0 (0%) in the ICE group and 14 (1.3%) in the no ICE group, *p* < 0.001; none of these patients received cardiac surgery but 4 (29%) required pericardiocentesis. The other periprocedural complications were not significantly different between the two groups (Table 2).

**Figure 2:**
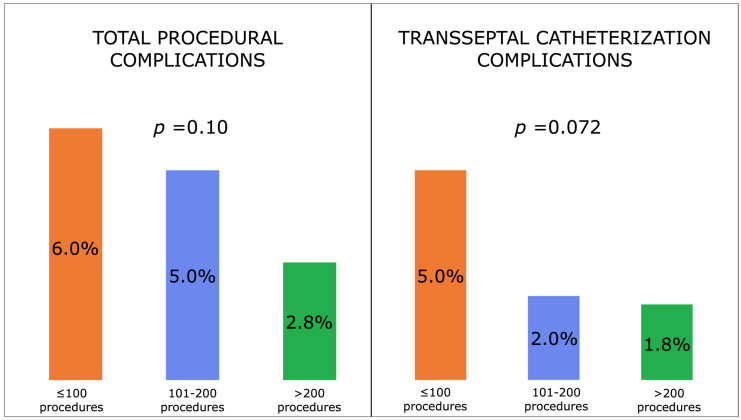
Total and transseptal catheterization-related procedural complications divided between the first 100, 101–200 and >200 procedures.

Dividing the procedures into chronological order of 1–100, 101–200 and >200, there were no statistically significant differences between the groups in periprocedural complications (6.0% vs. 5.0% vs. 2.8%, respectively, *p* = 0.10) and TSC complications (5.0% vs. 2.0% vs. 1.8%, respectively, *p* = 0.072). However, a trend toward lower complications was observed when the number of procedures was increasing (Figure 2).

The procedural effectiveness was 98%. An inability to perform the transseptal puncture and introduce the scaler catheter into the left atrium was observed in 41 procedures: 19 procedures in the ICE group and 22 procedures in the no ICE group (*p* = 0.57). Considering these 41 procedures, a total of 18 procedures produced complications, 1 in the ICE group and 17 in the no ICE group (*p* < 0.001), and 23 were forced to stop the procedure without further complications, 18 in the ICE group and 5 in the no ICE group (*p* = 0.02).

### Predictors of Complications

On univariate and multivariate analysis, age (OR 1,02 95% C.I 1.00–1.05; *p* = 0.036), TSC with the use of ICE (OR 0,27 95% C.I 0.15–0.46; *p* < 0.001) and AF ablation (OR 2.25 95% C.I 1.05–4.83; *p* = 0.037) were independent predictors of complications (Table 3). The independent predictors for TSC complications were, again, age (OR 1.03 95% C.I 1.01–1.06; *p* = 0.013) and TSC with the use of ICE (OR 0.24 95% C.I 0.11–0.49; *p* < 0.001) (Table 4). Furthermore, limiting the analysis to AF ablation, only the use of ICE was independently associated with a lower incidence of periprocedural complications (OR 0.34 95% C.I 0.19–0.60; *p* < 0.001) and TSC complications (OR 0.30 95% C.I 0.14–0.65; *p* < 0.001 (Table 5 and Table 6)).

## 4. Discussion

The main finding of this study was that the use of ICE significantly reduced periprocedural complications and TSC-related complications, with a relative risk reduction of 70%. Furthermore, the present findings show that the beneficial effect of ICE-guided procedures was independent of the learning curve. The other independent predictors of complications were advanced age and AF ablation. Age is a well-known factor in increasing procedural risk in most cardiac and non-cardiac interventions [9]. Ablation of the arrhythmic substrate of the AF (in comparison to the ablation of other left arrhythmias) was significantly associated with a higher incidence of complications. Greater tissue fragility in older patients and the greater procedural complexity of AF ablation can probably explain these observations [10].

Interestingly, in the last 5 years, supraventricular arrhythmias ablations increased, especially because of an increase in AF ablation procedures. This can be explained by the new recommended guidelines [11,12] that state AF ablation as one of the most effective procedures for a rhythm control strategy. Hand in hand, the use of ICE during left atrium procedures has increased from 8% to 92%.

The rate of periprocedural complications in the study population was 3.1%, in line with previous studies, and, in particular, was 1.7% in the ICE group and 4.7% in the no ICE group. Recently, Pimentel et al. reported in a series of more than 2000 patients a rate of complications that was 2.9% in the ICE group and 5.8% in the no ICE group, which is in line with our results [13].

In our cohort, the complications rate directly related to TSC (accidental puncture of aortic and atrial walls, pericardial effusion) fell to a value of 0.8%, equal to or less than previously published rates [1,2,3,4,5]. However, in our population, two common complications (aortic root puncture and atrial wall puncture) were not seen when using ICE.

All these results were confirmed by only analyzing AF ablation procedures. Of interest, age in this subgroup of patients was not independently related to complications. This may be due to the range of age being narrow, which may have homogenized the risk. An uninterrupted anticoagulation strategy, currently in class I in the ESC guidelines on AF management, was also associated with a significant reduction in global complications at univariate analysis but not independent in multivariate analysis where the only use of ICE was associated with lower complications. Considering the potential hemorrhagic risk of this strategy, the use of ICE could be of benefit by preventing accidental punctures of fragile structures. Even an infusion of heparin boluses before TSC, which in some studies has been associated with a lower incidence of intra-atrial thrombosis, could also be made safer by the use of ICE.

The impact of ICE on the incidence of TSC complications was not significant in a study by Swedish authors on 4690 procedures; however, it was used only in 0.5% of cases [14]. Our results, moreover, are in agreement with the data of previous series [9,13,15] and a large recently published US registry [7] on patients undergoing AF ablation that showed a significant reduction in periprocedural complications, in-hospital mortality and hospital stay in the ICE group, at the expense of a significant increase in costs. The shorter hospitalization times, according to the authors, were, however, able to make the procedure with ICE cost-effective.

Regarding the learning curve, we observed that there was a trend toward a higher number of periprocedural and TSC complications in the group of the first 100 procedures (1–100) and procedures 101–200 without, however, reaching statistical significance. This showed that experience alone is not enough to prevent complications, because cardiac anatomy is unpredictable. Therefore, the use of ICE is of paramount importance. The procedural efficiency, understood as the ability to complete TSC, was high (around 98%) in both groups of this study, although the complications arose significantly more often in the non-ICE group.

A recent meta-analysis [16] has shown that ICE is able to significantly reduce procedural times and fluoroscopy time and dose during the ablation of arrhythmias in the left atrium, without affecting the incidence of periprocedural complications. However, the latter finding has been derived from a very limited incidence (close to 0) of complications even in the group without the use of ICE, which differs from that reported by the larger registers mentioned above.

In conclusion, the additional cost of the ICE seems justified by the possibility of significantly reducing the incidence of complications and improving the procedural efficiency of TSC. Added to this is the possibility of highlighting and then treating the formation of any pericardial effusion, to visualize the esophagus during ablation and intra-atrial thrombus. In addition, the formation of microbubbles, visible with ICE, may be a frequent indication of excessive heating of the tissues during radiofrequency delivery and, finally, to select in certain cases the site of puncture of the oval fossa in relation to the type of procedure, with the possible benefit of the maneuverability of catheters [17]. Finally, the integration of the ICE with electroanatomical mapping systems has also proved very promising in the drastic reduction in scan times during TSC [18,19,20].

## 5. Limitations

The present study is retrospective, thus discounting the intrinsic limitations of this type of analysis. The time span covered is very long, with relative difficulty in completing the data collection. It was not possible, in fact, to evaluate the impact of the ICE on procedural times and on the times of fluoroscopy since such data were not available for all patients in the study. The data collection involved a limited number of centers with medium to high procedural volume, which, however, showed homogeneity in the execution of the TSC method. The use of ICE was not random but at the physician’s discretion and has become systematic from 2016 onwards. Complications not related to hospitalizations such as atrio-esophageal fistula and pulmonary vein stenosis were not reported. However, the present study focused on the role of ICE in reducing acute and TSC complications.

## 6. Conclusions

In this retrospective multicenter study, ICE significantly reduced periprocedural and TSC-related complications during electrophysiological procedures for the ablation of left atrial arrhythmias.

## Figures and Tables

**Table 1:** Characteristics of the population—1862 patients corresponding to 2181 procedures. ICE: intracardiac ecocardiography; AF: atrial fibrillation.

	Total Procedures *n* = 2181	ICE *n* = 1134	No ICE *n* = 1047	*p* Value
Age (years)	55 ± 14	59 ± 12	52 ± 16	<0.001
Male sex	1630 (75%)	834 (74%)	796 (76%)	0.19
Structural heart disease	395 (18%)	228 (20%)	167 (16%)	0.014
Left atrial diameter (mm)	43 ± 6	43 ± 6	43 ± 5	1.0
Left ventricular diameter (mm)	51 ± 4	51 ± 6	51 ± 3	1.0
Ejection fraction (%)	58 ± 7	58 ± 8	58 ± 5	1.0
AF ablation	1652 (76%)	1003 (88%)	649 (62%)	<0.001
Other ablation in left atrium	529 (24%)	131 (12%)	398 (38%)	<0.001
Redo	269 (12%)	152 (13%)	117 (11%)	0.13

**Table 2:** Procedural complications. ICE: intracardiac ecocardiography.

	Total Procedures *n* = 2181	ICE Group *n* = 1134	No ICE Group *n* = 1047	*p* Value
Periprocedural complications:	67 (3.1%)	19 (1.7%)	48 (4.6%)	<0.001
Transseptal cathetherization complications	42 (1.5%)	10 (0.9%)	32 (3.1%)	<0.001
- Vascular access complications, total:	20 (0.9%)	5 (0.4%)	15 (1.4%)	0.028
- Groin hematoma, total:		4	14	
- Major groin hematoma		1	3	
- Retroperitoneal hematoma		1	1	
- Arterio-venous fistula		0	1	
- Aortic root puncture, total:	8 (0.4%)	0 (0%)	8 (0.8%)	0.003
- No consequences		0	2	
- Pericardiocentesis		0	2	
- Cardiac surgery		0	4	
- Atrial wall injury, total:	14 (0.6%)	0 (0%)	14 (1.3%)	<0.001
- Contrast in pericardium		0	10	
- Pericardiocentesis		0	4	
- Cardiac perforation		0	0	
- Pericarditis and/or pericardial effusion	12 (0.6%)	6 (0.5%)	6 (0.6%)	0.89
- Thromboembolism, total:	7 (0.3%)	5 (0.4%)	3 (0.3%)	0.51
- Transient ischemic attack	2	1	1	
- Stroke	4	3	1	
- Systemic embolism	1	0	1	
- Acute coronary injury	6 (0.3%)	2 (0.2%)	4 (0.4%)	0.69
- Hemidiaphragm paresis	1	1 (0.1%)	0 (0%)	0.34
- Death	0	0	0	1.00

**Table 3:** Predictors of total procedural complication. ICE: intracardiac ecocardiography; AF: atrial fibrillation.

Variable	Univariate Analysis		Multivariate Analysis	
	Odds Ratio (95% CI)	*p* Value	Odds Ratio (95% CI)	*p* Value
ICE group	0.35 (0.21-0.62)	<0.001	0.27 (0.15-0.46)	<0.001
Age (for one year increase)	1.02 (1.00-1.04)	0.020	1.02 (1.00–1.05)	0.036
AF ablation	2.10 (1.03-4.27)	0.040	2.25 (1.05–4.83)	0.037
Structural heart disease	0.99 (0.52-1.86	0.97		
Reference >200 procedures		0.11		
Procedures 1–100	2.19 (0.92–5.22)			
Procedures 101–200	1.81 (0.71–4.62)			

**Table 4:** Predictors of TSC complication. ICE: intracardiac ecocardiography; AF: atrial fibrillation; TSC: transseptal catheterization.

Variable	Univariate Analysis		Multivariate Analysis	
	Odds Ratio (95% CI)	*p* Value	Odds Ratio (95% CI)	*p* Value
ICE group	0.28 (0.14–0.58)	0.001	0.24 (0.11–0.49)	<0.001
Age (for one year increase)	1.02 (1.00–1.05)	0.088	1.03 (1.01–1.06)	0.013
AF ablation	1.18 (0.56–2.48)	0.67		
Structural heart disease	0.99 (0.52–1.86	0.97		
Reference >200 procedures		0.090		
Procedures 1–100	2.93 (1.12–7.64)			
Procedures 101–200	1.13 (0.27–4.79)			

**Table 5:** Predictors of total procedural complications for AF ablation procedure. ICE: intracardiac ecocardiography; AF: atrial fibrillation.

Variable	Univariate Analysis		Multivariate Analysis	
	Odds Ratio (95% CI)	*p* Value	Odds Ratio (95% CI)	*p* Value
ICE group	0.30 (0.17–0.53)	<0.001	0.34 (0.19–0.60)	<0.001
Age (for one year increase)	1.01 (0.98–1.04)	0.45		
Uninterrupted anticoagulant	0.24 (0.08–0.79)	0.018	0.33 (0.10–1.08)	0.067
Structural heart disease	0.77 (0.39–1.49)	0.43		
Reference >200 procedures		0.36		
Procedures 1–100	1.64 (0.64–4.22)			
Procedures 101–200	1.70 (0.66–4.37)			

**Table 6:** Predictors of TSC complications for AF ablation procedure. ICE: intracardiac ecocardiography; AF: atrial fibrillation; TSC: transseptal catheterization.

Variable	Univariate Analysis		Multivariate Analysis	
	Odds Ratio (95% CI)	*p* Value	Odds Ratio (95% CI)	*p* Value
ICE group	0.27 (0.13–0.58)	0.001	0.30 (0.14–0.65)	0.002
Age (for one year increase)	1.01 (0.98–1.05)	0.45		
Uninterrupted anticoagulant	0.29 (0.07–1.24)	0.095	0.41 (0.10–1.77)	0.41
Structural heart disease	0.58 (0.22–1.52)	0.27		
Reference >200 procedures		0.30		
Procedures 1–100	2.34 (0.80–6.83)			
Procedures 101–200	1.18 (0.28–5.06)			

## Data Availability

The data underlying this article will be shared on reasonable request to the corresponding author.

## Appendix A

1. Cardiology Unit, S. Maria Nuova Hospital, 42100 Reggio Emilia, Italy
2. Arrhythmologic Center, Lavagna Hospital, 16033 Lavagn, Italy
3. Guglielmo da Saliceto Hospital, 29121 Piacenza, Italy
4. Cardiogy Unit, University Hospital of Ferrara, 8-44124 Cona, Italy
5. Electrophysiology and Cardiac Pacing, Division of Cardiology, Cardio-Thoracic Department, University Hospital of Verona, 1-37126 Verona, Italy
